# Conservation of Non-Pest Whiteflies and Natural Enemies of the Cabbage Whitefly *Aleyrodes proletella* on Perennial Plants for Use in Non-Crop Habitats

**DOI:** 10.3390/insects12090774

**Published:** 2021-08-29

**Authors:** Sebastian Laurenz, Rainer Meyhöfer

**Affiliations:** Section Phytomedicine, Institute of Horticultural Production Systems, Leibniz Universität Hannover, Herrenhäuser Straße 2, 30419 Hannover, Germany; meyhoefer@ipp.uni-hannover.de

**Keywords:** *Aleyrodes lonicerae*, *Encarsia tricolor*, alternative host/prey, conservation biological control, field margin, functional biodiversity, habitat management, landscape ecology, parasitoids, predators

## Abstract

**Simple Summary:**

The cabbage whitefly *Aleyrodes proletella* is a major insect pest of many cabbage crops. Natural enemies, in particular *Encarsia tricolor* as well as different hoverfly larvae and spiders, do not decrease pest populations sufficiently. The objective of this study is to promote local natural enemy populations by permanently establishing a non-pest whitefly species, which is an alternative host and additional food source when *A. proletella* is scarce or even absent. Therefore, the perennial abundance of the non-pest honeysuckle whitefly *Aleyrodes lonicerae* and natural enemies on different plants were evaluated in the open field. Wood avens *Geum urbanum* was the best host plant for *A. lonicerae* in terms of reproduction and overwintering. Most *E. tricolor* and spiders were also found on this plant species. In the future, *G. urbanum* might be used in non-crop habitats to increase natural enemy abundances in the agricultural landscape and decrease damage caused by *A. proletella* on adjacent cabbage plants.

**Abstract:**

*Aleyrodes proletella* causes severe economic damage to several *Brassica* crops. Its naturally occurring enemies often immigrate late in the season or appear in low numbers on cabbage. This field study aims to permanently increase the local abundance of *A. proletella*’s natural enemies by providing the non-pest whitefly *Aleyrodes lonicerae* as an alternative and overwintering host/prey. Therefore, the population dynamics of natural enemies on different perennial herbaceous plants pre-infested with *A. lonicerae* were determined at two field locations over two winter periods. Most *A. lonicerae* colonized (on average 166.22 puparia per m²) and overwintered (342.19 adults per m²) on wood avens *Geum urbanum*. Furthermore, the abundance of *A. proletella* main parasitoid *Encarsia tricolor* (28.50 parasitized puparia per m²) and spiders (12.13 per m²) was 3–74 times and 3–14 times higher, respectively, on *G. urbanum* compared to the other experimental plants. Conclusively, *G. urbanum* pre-infested with *A. lonicerae* permanently promoted natural enemies of *A. proletella* by serving as shelter, reproduction, and overwintering habitat. A potential implementation of *G. urbanum* in conservation biological control strategies (e.g., tailored flower strips, hedgerows) against *A. proletella* are discussed and suggestions for future research are given.

## 1. Introduction

*Aleyrodes proletella* Linnaeus (Hemiptera: Aleyrodidae) has become a major pest on *Brassica* crops in different parts of the world during the past decades [1,2,3,4,5]. Its parasitoid *Encarsia tricolor* Förster (Hymenoptera: Aphelinidae) is distributed over large parts of Europe up to Russia and Northern Africa and is regarded as the most important natural enemy of *A. proletella* in Central Europe next to hoverfly larvae, coccinellids, and spiders among others [5,6,7,8,9,10,11,12,13,14,15,16,17].

Nevertheless, the migration of *E. tricolor* from its overwintering sites into cabbage crops often occurs too late in the season and insufficiently in numbers to regulate *A. proletella* populations substantially [18,19]. In close distance to the crop, *E. tricolor* and also other natural enemies often lack appropriate shelter, overwintering, and reproduction habitats that permanently provide natural enemies with alternative hosts and prey even if *A. proletella* is absent. In Europe, at least 44 naturally occurring whitefly species may potentially serve as alternative hosts for *E. tricolor* next to *A. proletella* [11,16]. However, most of these whitefly species are usually present only in relatively low numbers in the field [17].

The abundance of alternative whitefly hosts and their host plants in the agricultural landscape might be enhanced temporarily for one growing season. An annual banker plant system comprising *Trialeurodes vaporariorum* Westwood (Hemiptera: Aleyrodidae) as alternative host and *E. tricolor* on Hokkaido squash was already successfully tested in the field [20]. The enhanced abundance of alternative whitefly hosts increased hoverfly larvae abundance by 62% and *A. proletella* parasitism rates by 50% on adjacent cabbage crops, which led to a decrease of *A. proletella* populations by up to 26%. However, *T. vaporariorum* does not survive Central European winters in the open field and therefore does not serve as an overwintering host for *E. tricolor* [18].

A more long-term option to increase the local abundance of natural enemies in the agricultural landscape is to enhance the number of overwintering hosts as part of a conservation biological control strategy. *E. tricolor* overwinters as immature developmental stages inside whitefly nymphs [17,18]. *A. proletella* (e.g., on winter crops like oilseed rape) or naturally occurring non-pest whitefly species may play a role [21,22,23]. An increase of the latter could be achieved by a targeted composition of non-crop areas like field margins, hedgerows, or flowering strips with evergreen perennial host plants of non-pest whiteflies [24]. Potential plant-whitefly combinations facilitating natural enemies of *A. proletella* in Central Europe might for instance be *Lonicera* spp. (Caprifoliaceae) and several herbaceous host plants for *A. lonicerae*, *Fraxinus* spp. (Oleaceae) for *Siphoninus phillyreae* Haliday (Hemiptera: Aleyrodidae) or *Viburnum* spp. (Adoxaceae) for *Aleurotuba jelinekii* Frauenfeld (Hemiptera: Aleyrodidae) [5,14,25,26].

In particular, the polyphagous and widespread in Europe *A. lonicerae* has been evaluated as a promising host for *E. tricolor* on different host plants under controlled conditions. Thus, the present study aimed to identify perennial herbaceous host plants of *A. lonicerae* for their suitability to permanently conserve and promote *E. tricolor* and other natural enemies of *A. proletella* in the field.

## 2. Materials and Methods

Same experiments were installed at two locations in Germany, i.e., Hannover (52°23′39.0″ N 9°42′18.2″ E) and Sarstedt (52°14′39.8″ N 9°49′13.5″ E) in August 2015. In Hannover, the mean temperature was 11.27°C, and the precipitation 562.6 mm during the experimental period. The mean temperature and precipitation in Sarstedt were 4.63°C and 476.13 mm, respectively.

### 2.1. Inoculation of Plants with Alternative Hosts

Experimental plants, i.e., European columbine *Aquilegia vulgaris* (Ranunculaceae), peach-leaved bellflower *Campanula persicifolia* (Campanulaceae), wild strawberry *Fragaria vesca* and wood avens *Geum urbanum* (both Rosaceae), were selected based on the survival, development, and reproduction of *A. lonicerae*, *A. proletella* and their common parasitoid *E. tricolor* in a previous laboratory study [20]. They were sown from untreated seeds and grown separated by species under four gauze tents (length: 3 m, width: 1.5 m, height: 2 m; 500 plants per tent) in a greenhouse. Plants were inoculated with *A. lonicerae* when one to three true leaves were fully expanded. Adult *A. lonicerae* derived from the main rearing established on *Aegopodium podagraria* (ground elder) at the Section Phytomedicine, Institute of Horticultural Production Systems, Leibniz Universität Hannover, Germany. For inoculation, 1200 adult *A. lonicerae* females (2.4 females per plant) and 300 males were evenly released under each gauze tent. Females were allowed to oviposit for 35 days before plants were transferred to the field. At planting in August 2015, 6.91 ± 1.20 puparia (mean ± SE) were present on each plant.

### 2.2. Experimental Set-Up

Experimental plots (1.6 m × 1.6 m) were arranged in line in a randomized block design with three blocks and five treatments, i.e., *A. vulgaris*, *C. persicifolia*, *F. vesca*, *G. urbanum,* and an equal mixture of all four plant species, per location. Plants (64 per plot) were planted with 20 cm distance to each other and plots were separated by 40 cm bare soil. Other naturally occurring vegetation in the plots was regularly removed if needed. All assessments were done on four randomly selected plants per plot from 16 December 2015 to 14 December 2016. Border plants were not assessed and one plant of each species was selected in the mixed treatment. Every four weeks, the number of whitefly puparia (last nymphal stage) and parasitized whitefly puparia as well as the number of other herbivores and predatory arthropods were counted per plant in the field (14 assessment dates in total). Additionally, three leaves per plot with unparasitized and parasitized puparia were sampled every four months (4 samplings in total), transferred to gauze bags, and incubated at room temperature in order to identify the emerging adult whiteflies and parasitoids, respectively, to species level. The number of overwintering adult whiteflies per plant was determined twice, i.e., in December of both years.

### 2.3. Statistics

Data were statistically analyzed with R version 4.0.5 for each location separately [27]. Plots and data for descriptive statistics were provided by the packages ‘car’ and ‘FSA’ (Fisheries Stock Analysis), respectively [28,29]. All assessed data per plant were calculated to the number of individuals per m² for each plot before statistical analysis. Differences between treatments in terms of the number of whitefly puparia and parasitized whitefly puparia, other herbivores, and predators were determined with generalized linear mixed-effects models (glmer) fit by maximum likelihood (package ‘lme4’) [30]. Count data were fitted with negative binomial models (glmer.nb) and a log link function to deal with overdispersion [31]. Linear mixed-effects models (lmer) fit by restricted maximum likelihood (REML) were applied to compare the treatments with each other regarding the number of overwintering adult *A. lonicerae* (package ‘lme4’) [30]. The package ‘blmeco’ determined dispersion parameters [32]. Treatment (*A. vulgaris*, *C. persicifolia*, *F. vesca*, *G. urbanum*, mix) and assessment date were set as explanatory variables. Data were repeatedly collected from the same plots. Thus, an identification number was assigned to each plot, which was taken as a random effect to account for temporal non-independence. The Akaike information criterion (AIC) was used for model evaluation, i.e., the model with the lowest AIC value (highest accuracy) was chosen for each response variable to compute an analysis of deviance table (ANOVA function). Tukey post hoc was applied for multiple comparisons of means to determine differences between treatments (package ‘multcomp’) [33].

## 3. Results

### 3.1. Herbivore Population Development

The amount of overwintering adult *A. lonicerae* was different on experimental plant species in Hannover (*χ*^2^ (4, *N* = 30) = 16.47, *p* = 0.003) as well as in Sarstedt (*χ*^2^ (4, *N* = 30) = 61.46, *p* < 0.001). *G. urbanum* served as the best overwintering host for *A. lonicerae* adults at both locations. In Hannover, the average number of overwintering *A. lonicerae* per m² over the two experimental winters was far higher on *G. urbanum* (380.21 ± 164.27) than on *C. persicifolia* (2.08 ± 1.32) (*p* < 0.001). There was no difference to the treatments *A. vulgaris* (233.33 ± 113.99), *F. vesca* (172.92 ± 82.74), and mix (245.83 ± 124.74) (*p* > 0.05). In Sarstedt, more adult *A. lonicerae* adults overwintered on *G. urbanum* (304.17 ± 65.60) than on *F. vesca* (184.38 ± 58.85), *A. vulgaris* (102.08 ± 46.85) and *C. persicifolia* (23.96 ± 14.29) (*p* = 0.02, *p* < 0.001 and *p* < 0.001, respectively). Furthermore, less *A. lonicerae* adults were observed on *A. vulgaris* and *C. persicifolia* compared to the mixed treatment (230.21 ± 66.78) (*p* = 0.01 and *p* < 0.001, respectively). *F. vesca* was a better overwintering host for *A. lonicerae* than *C. persicifolia* (*p* < 0.001). No other differences between plant species were detected (*p* > 0.05).

**Figure 1:**
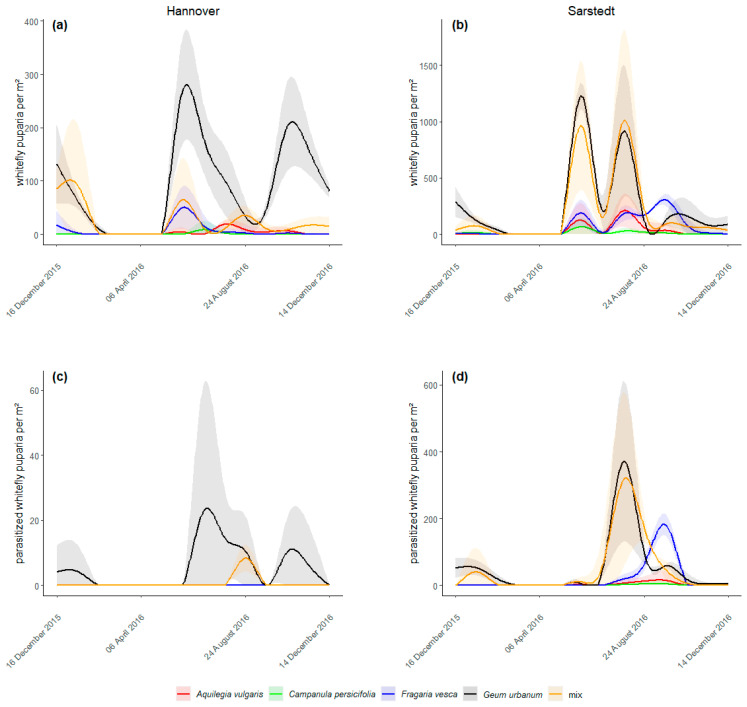
Population dynamics of whitefly puparia (**a**,**b**) and parasitized whitefly puparia (**c**,**d**) in Hannover and Sarstedt over the experimental period. The shaded areas represent the 95% confidence intervals.

The abundance of *A. lonicerae* puparia differed between the treatments in Hannover (Figure 1a, *χ*^2^ (4, *N* = 210) = 186.08, *p* < 0.001) and in Sarstedt (Figure 1b, *χ*^2^ (4, *N* = 210) = 84.47, *p* < 0.001). The average number of puparia per m² over the entire experimental period ranged in Hannover from 0.74 ± 0.61 (*C. persicifolia*) to 91.52 ± 14.10 (*G. urbanum*) and in Sarstedt from 10.57 ± 3.83 (*C. persicifolia*) to 240.92 ± 58.57 (*G. urbanum*). At both locations, *G. urbanum* harbored more *A. lonicerae* puparia than any other plant species (all *p* < 0.001). More statistical differences are shown in Table 1.

No whitefly species other than *A. lonicerae* (e.g., *A. proletella*) was observed on any experimental plant at any assessment date. The numbers of other herbivores (i.e., aphids) were insufficient for statistical analysis.

### 3.2. Abundance of Naturally Occurring Enemies

All samples of parasitized whitefly puparia taken from the experimental plants were identified as *E. tricolor*. At both locations the abundance of parasitized puparia differed between treatments (Hannover: Figure 1c, *χ*^2^ (4, *N* = 210) = 37.06, *p* < 0.001; Sarstedt: Figure 1d, *χ*^2^ (4, *N* = 210) = 152.20, *p* < 0.001). In Hannover, parasitized puparia were only on *G. urbanum* (on average 5.21 ± 1.78 individuals per m²) and in the mixed treatment (0.60 ± 0.36), i.e., no whitefly parasitism on the other plant species was recorded. The number of parasitized puparia was higher on *G. urbanum* than in all other treatments (all *p* < 0.001). In Sarstedt, the average number of parasitized puparia per m² ranged from 0.74 ± 0.44 (*C. persicifolia*) to 51.79 ± 16.30 (*G. urbanum*). Statistical differences are given in Table 1.

Spider abundance was also different between the treatments in Hannover (*χ*^2^ (4, *N* = 210) = 53.02, *p* < 0.001) as well as in Sarstedt (*χ*^2^ (4, *N* = 210) = 23.30, *p* < 0.001) with *G. urbanum* inhabiting the most spiders at both locations. In Hannover, more spiders per m² were found on *G. urbanum* (on average 16.52 ± 3.81) and the mixed treatment (7.29 ± 1.40) compared to *A. vulgaris* (1.64 ± 0.71), *F. vesca* (1.34 ± 0.40) and *C. persicifolia* (1.19 ± 0.61) (all *p* < 0.01). No other differences were determined (*p* > 0.05). In Sarstedt, *G. urbanum* (7.74 ± 1.56), the mix treatment (4.32 ± 0.89) and *C. persicifolia* (2.98 ± 0.78) sheltered more spider per m² than *F. vesca* (0.89 ± 0.34; *p* < 0.001, *p* = 0.02 and *p* = 0.02, respectively). Additionally, *G. urbanum* had more spiders than *A. vulgaris* (1.94 ± 0.62; *p* = 0.04). The number of predators other than spiders (i.e., hoverfly larvae, ladybeetles, predatory bugs, lacewing larvae, predatory flies, and gall midge larvae) were insufficient and could not be analyzed statistically.

## 4. Discussion

This two-year field study evaluated perennial host plants of *A. lonicerae* for the purpose of the conservation biological control of *A. proletella*. Populations of alternative hosts/prey (*A. lonicerae*) and natural enemies (*E. tricolor* and spiders) of *A. proletella* established, overwintered, and permanently settled in the highest numbers on *G. urbanum*.

The parasitoid *E. tricolor* but also spiders are next to hoverfly larvae among the most important natural enemies of *A. proletella* [15]. An improvement of resilience of *E. tricolor* by annual banker plants has already been shown to increase parasitism rates and decrease *A. proletella* populations on cabbage [20]. More research is needed to investigate the impact of a permanent increase in local natural enemy abundance by *G. urbanum* in field margins on *A. proletella* populations on adjacent cabbage crops. Furthermore, it needs to be evaluated if pre-infestation with *A. lonicerae* (done in the current study) is necessary, or if the natural infestation can build up a suitable reservoir.

Hoverfly larvae might not have been affected significantly in this study, because hoverfly adults depend on floral resources from suitable flowers, which were hardly available [34,35]. Thus, a combination of *G. urbanum* with suitable flowering plants might lead to even better conservation of the natural enemies of *A. proletella* and should be addressed in future research.

Among all experimental plant species, *G. urbanum* increased *A. lonicerae* and natural enemy abundance at both experimental locations most, even in the second winter of the study. However, plants in Sarstedt inhabited more *A. lonicerae* and *E. tricolor* than the ones in Hannover. Soil analyses at both locations revealed that the soil in Hannover lacked nitrogen (N), potassium (K), and magnesium (Mg) resulting in generally larger and more vital plants in Sarstedt. Therefore, the plants in Sarstedt represented a qualitatively and quantitatively better nutritional source and thus may have benefited both, *A. lonicerae* and its parasitoid *E. tricolor* [36]. Next to plant nutrition, local differences in climatic parameters (e.g., on average 7°C warmer and in total 87 mm more precipitation in Hannover compared to Sarstedt during the entire experimental period), vegetation or additional food sources are further potential factors that could have influenced the abundances and colonization with herbivores and natural enemies at the two locations.

None of the investigated plant species served as a host plant for *A. proletella*. This whitefly pest might be able to survive and successfully reproduce to a certain extent, if caged onto these plants in the laboratory [20]. However, no adult *A. proletella* was observed on any plant species at any location and at any assessment date in the present field study, although cabbage plants with *A. proletella* populations were present in about 50 m distance to the experimental plots at both locations.

No *A. lonicerae* puparia and thus no parasitism was observed at either location from March to May 2016. Only whitefly adults that either overwintered or recently emerged from overwintered nymphs, first deposited eggs and already young nymphs (potential parasitism not visible in the field), and *E. tricolor* adults emerged from overwintered immature stages were present during this period [18]. Due to low spring temperatures, deposited whitefly eggs needed until 1st June before they developed into puparia (Figure 1a,b). First parasitized puparia appeared a few weeks later by the end of June (Figure 1c,d).

The number of *E. tricolor* pupae, i.e., dark whitefly puparia, during winter months was relatively low. This parameter was determined to estimate the abundance of whitefly parasitoids on the plants during the entire experimental period. However, this parameter actually underestimates the number of overwintering *E. tricolor*, since most *E. tricolor* overwinter as eggs or young larvae inside whitefly nymphs without showing morphological differences [18].

In conclusion, *G. urbanum* (pre-)infested with *A. lonicerae* is able to permanently increase the local abundance of natural enemies of *A. proletella*. Future research might focus on the added effect of *G. urbanum* in non-crop habitats (e.g., field margins, hedgerows, flowering strips) on the populations of natural enemies and *A. proletella* on adjacent cabbage crops and on cabbage yield. More general, similar conservation biological control strategies using alternative hosts to permanently promote natural enemy populations can be established targeting other important horticultural or agricultural pests. Finally, the conservation of non-pest herbivores in non-crop habitats might not only suppress mass outbreaks of pest populations and decrease insecticide applications but might also counteract biodiversity loss in agricultural landscapes.

## Figures and Tables

**Table 1:** Mean numbers of non-parasitized and parasitized whitefly puparia per m² (mean ± SE) per plant species over the entire experimental period at the two locations (in brackets). Different small letters indicate statistical differences between plant species (α = 0.5).

Parameter (Location)	Mean Number of Individuals per m² over Experimental Period				
	*A. vulgaris*	*C. persicifolia*	*F. vesca*	*G. urbanum*	mix
whitefly puparia (Hannover)	2.98 ± 0.98 ^b^	0.74 ± 0.61 ^a^	6.70 ± 2.62 ^b^	91.52 ± 14.10 ^d^	25.15 ± 6.56 ^c^
whitefly puparia (Sarstedt)	31.25 ± 11.76 ^a^	10.57 ± 3.83 ^a^	69.20 ± 15.95 ^b^	240.92 ± 58.67 ^c^	193.30 ± 59.45 ^bc^
parasitized puparia (Hannover)	0.00 ± 0.00 ^a^	0.00 ± 0.00 ^a^	0.00 ± 0.00 ^a^	5.21 ± 1.78 ^b^	0.60 ± 0.36 ^a^
parasitized puparia (Sarstedt)	2.53 ± 1.28 ^ab^	0.74 ± 0.44 ^a^	19.64 ± 7.61 ^b^	51.79 ± 16.30 ^d^	46.58 ± 16.22 ^c^

## Data Availability

The data presented in this study are openly available in LUH-Projekt Seafile at https://doi.org/10.25835/0021354 (accessed on 16 August 2021) [37].
